# Hunting for the beat in the body: on period and phase locking in music-induced movement

**DOI:** 10.3389/fnhum.2014.00903

**Published:** 2014-11-07

**Authors:** Birgitta Burger, Marc R. Thompson, Geoff Luck, Suvi H. Saarikallio, Petri Toiviainen

**Affiliations:** Department of Music, Finnish Centre for Interdisciplinary Music Research, University of JyväskyläJyväskylä, Finland

**Keywords:** music-induced movement, dance, motion capture, entrainment, period locking, phase locking

## Abstract

Music has the capacity to induce movement in humans. Such responses during music listening are usually spontaneous and range from tapping to full-body dancing. However, it is still unclear how humans embody musical structures to facilitate entrainment. This paper describes two experiments, one dealing with period locking to different metrical levels in full-body movement and its relationships to beat- and rhythm-related musical characteristics, and the other dealing with phase locking in the more constrained condition of sideways swaying motions. Expected in Experiment 1 was that music with clear and strong beat structures would facilitate more period-locked movement. Experiment 2 was assumed to yield a common phase relationship between participants' swaying movements and the musical beat. In both experiments optical motion capture was used to record participants' movements. In Experiment 1 a window-based period-locking probability index related to four metrical levels was established, based on acceleration data in three dimensions. Subsequent correlations between this index and musical characteristics of the stimuli revealed pulse clarity to be related to periodic movement at the tactus level, and low frequency flux to mediolateral and anteroposterior movement at both tactus and bar levels. At faster tempi higher metrical levels became more apparent in participants' movement. Experiment 2 showed that about half of the participants showed a stable phase relationship between movement and beat, with superior-inferior movement most often being synchronized to the tactus level, whereas mediolateral movement was rather synchronized to the bar level. However, the relationship between movement phase and beat locations was not consistent between participants, as the beat locations occurred at different phase angles of their movements. The results imply that entrainment to music is a complex phenomenon, involving the whole body and occurring at different metrical levels.

## Introduction

Music has the capacity to induce movements in humans (Lesaffre et al., 2008; Keller and Rieger, 2009). Such movement responses during music listening are usually spontaneous and can range from tapping along with the music to full-body dance movements. Typically, music-induced movement is found to be both organized and coordinated; for instance, people mimic instrumentalists' gestures or entrain with the pulse of the music (Godøy et al., 2006; Leman and Godøy, 2010). Moreover, Leman (2007, p. 96) suggests, “Spontaneous movements [to music] may be closely related to predictions of local bursts of energy in the musical audio stream, in particular to the beat and the rhythm patterns.” Such bodily entrainment to music is the topic of the present paper, in particular how humans embody periodicities in music, which musical characteristics facilitate periodic movement, and what kind of phase relationships exist between movements and musical beat locations. Entrainment refers to the temporal attunement of two (or more) independent organisms resulting in coordinated rhythmic behavior (Large, 2000; Phillips-Silver et al., 2010; Clayton, 2012). For instance, a human can entrain with another human and/or with an external (musical) stimulus—periodic oscillations (usually a beat structure) that enable a listener to sustain a stable periodic pattern and synchronize movements (Large, 2000). Besides in humans, entrainment to rhythmic music has been found in vocal learning nonhuman species, such as the cockatoo Snowball (Patel et al., 2008).

Musical beats often occur at regular temporal intervals, giving rise to a percept of a pulse in the music and a subjective sense of periodicity. By subdividing this basic pulse into smaller units, as well as grouping pulses into larger cycles, a metrically interlocked grid with subjectively stronger and weaker events is created (Parncutt, 1994). These subdivisions are hierarchically organized, with different temporal levels typically related by periods with simple integer ratios (Palmer and Krumhansl, 1990). Most frequently for music in duple meter (every second beat is accentuated), four metrical levels are of interest: half the beat period (i.e., eighth note level), the beat period (i.e., quarter note or tactus level), double the beat period (i.e., half note level), and four times the beat period (i.e., whole note or bar level). Moreover, the theory of *dynamic attending* (e.g., Jones, 1976; Drake et al., 2000) puts forward that humans, when listening to a complex auditory sequence, spontaneously concentrate on events happening at a medium rate (called *referent level*, mostly correspondent to the tactus/beat level), and get locked or *attuned* to this periodicity, but are also able to dynamically shift attention to events happening at either higher or lower metrical levels and get attuned to these periodicities instead. This process is called *focal attending* and is based on *expectancy schemes* (corresponding to the metrical structure) that enable anticipation of consecutive events belonging to one or more metrical levels. Humans tend to easily perceive and process these metrical structures and often respond by spontaneously entraining with them, predicting the temporal structure and adjusting the motor output to the sensory input (Fraisse, 1982; Arom, 1991). Furthermore, most cultures have developed coordinated dance movements to rhythmically predictable music (with a pulse perceived as being periodic) (Nettl, 2000), that are seen as elements crucial to most social and collective human behavior (Brown et al., 2000).

Human capabilities to spontaneously synchronize with a musical beat have been extensively studied by employing various (finger) tapping paradigms (for reviews on past and current research, see Repp, 2005; Repp and Su, 2013). Ranging from tapping to metronomes to beat-finding in complex music (e.g., Drake et al., 2000; Snyder and Krumhansl, 2001; Large et al., 2002; Toiviainen and Snyder, 2003; Keller and Repp, 2004), literature suggests that the human ability of finding and entraining to (musical) beats is spontaneous and accurate, occurring within periods between 300–900 ms (Fraisse, 1982; Parncutt, 1994; van Noorden and Moelants, 1999), and being most regular for periods around 400–500 ms (Collyer et al., 1992). This as well as results from free tapping experiments, which found that the majority of participants tapped at a rate of around 600 ms (Fraisse, 1982), gave rise to suggesting around 500 ms—or 120 beats per min (bpm)—as the *preferred tempo*: the rate at which tempo perception is considered to be optimal and most natural (Fraisse, 1982; Moelants, 2002). Furthermore, spontaneous and preferred tempo has been studied in other fields related to repeated motor activities. In walking, for example, a spontaneous duration of steps of about 500–550 ms has been found (Murray et al., 1964; MacDougall and Moore, 2005). Fraisse (1982) suggested walking to be “a fundamental element of human motor activity” (p. 152) and could therefore serve as an origin of preferred tempo perception. Results from a study investigating walking to music (Styns et al., 2007) further support the notion of a preferred tempo at around 110–120 bpm.

In relation to cyclical movement, the phenomenon of *anchoring* has been reported (Beek, 1989); this refers to cyclic movements being timed with respect to a distinct point in each cycle, at which the spatial and temporal variability of the executed movement decreases. The anchor points can be locations of movement reverses or endpoints (Roerdink et al., 2008), though they can also occur at other characteristic points during the cycle (i.e., the ball release in juggling; Beek, 1989). They might therefore serve as “intentional attractors,” or “organization centers,” having a stabilizing function for the cyclic movement (Beek, 1989).

A recent study conducted by Janata et al. (2012), in which participants were asked to tap to music, found that participants not only moved the finger/hand, but also other body parts, such as feet and head. Additionally, the tapping condition (isochronous vs. free tapping) influenced the amount of movement: the more “natural” the tapping condition, the more movement was exhibited. Su and Pöppel (2012) studied if moving along with rhythms changes beat detection. Given the task of tapping in synchrony with the beat of non-isochronous rhythms as soon after the stimulus onset as possible, half the participants were asked to sit still, whereas the other half was told to move along when the stimuli started. Musicians performed equally well in both conditions (correct synchronization in 88% of the trials for movement condition, 78% for sitting condition); however, moving non-musicians found a stable beat in 79% of the cases, whereas sitting non-musicians only in 29% of the cases. The moving non-musicians were also faster in finding a beat than the non-moving non-musicians. These results suggest that musicians, as opposed to non-musicians, might have some kind of effective internal motor simulation related to beat perception, whereas non-musicians have to actually execute the movement.

Neurobiological studies indicate links between rhythmic (and beat) components of music and movement, as several connections between auditory, and motor systems in the brain have been observed (for overviews see Zatorre et al., 2007; Patel and Iversen, 2014). Grahn and Brett (2007) postulated that beat reproduction is mediated by motor areas. Moreover, Grahn and Rowe (2009) have observed activity in the motor system even without actual movement while listening to music. They performed an fMRI study, in which people were instructed not to move when perceiving the beat, and found different levels of activity and connectivity in putamen (basal ganglia), premotor cortex (PMC), supplementary motor area (SMA), and auditory cortex for the different types of stimuli. Chen et al. (2009) ran a study including three conditions, passive listening, anticipation of tapping, and actual tapping. They reported activity in different motor areas, such as PMC, SMA, and cerebellum, for all three conditions. However, the active part of the PMC was dependent on the condition: during passive listening, only the mid-PMC was active, while in the other two conditions the ventral PMC was active as well, and only in the tapping task the dorsal PMC was activated. However, this finding is at odds with results of Bengtsson et al. (2009), who located activation in the dorsal PMC—among other motor related areas—in a purely passive listening task without any movement intention. Stupacher et al. (2013) furthermore, established links between perceived groove and motor activity in the brain. Besides brain studies, also behavioral studies suggest links between movement/body and rhythm/beat aspects in music: Phillips-Silver and Trainor (2008) showed that especially head movements were found to bias metrical encoding of rhythm and meter perception. Moreover, Trainor et al. (2009) discovered that galvanic stimulation of the vestibular system could be used to disambiguate an ambiguous metric pattern. Todd et al. (1999) claimed that pulse perception inevitably requires motor system activity, since pulse is an inherently sensorimotor phenomenon, while Todd et al. (2007) found that 16% of variation in preferred beat rate could be predicted from anthropometric factors. Such findings and studies have led to the assumption that there is a predisposition for movement when listening to music and that humans furthermore prefer music that facilitates entrainment and respond to it with movement (Madison et al., 2011).

However, despite the considerable amount of literature on beat perception and tapping and synchronization to musical beats, far less studies have been investigating actual body movement in that context. Zentner and Eerola (2010) investigated infants' ability to bodily synchronize with musical stimuli, finding that infants showed more rhythmic movement to music and metrical stimuli than to speech, suggesting a predisposition for rhythmic movement to music and other metrical regular sounds. Eerola et al. (2006) studied toddlers' corporeal synchronization to music, finding three main periodic movement types being at times synchronized with the pulse of the music. Toiviainen et al. (2010) investigated how music-induced movement exhibited pulsations on different metrical levels, and showed that eigenmovements of different body parts were synchronized with different metrical levels of the stimulus. Naveda and Leman (2010) studied how the metric hierarchy in Samba and Charleston is represented in repetitive gestures of professional dancers. Van Dyck et al. (2013) found that an increased presence of the bass drum tends to increase listeners' spontaneous movements. Burger et al. (2013) could further show that certain beat- and rhythm-related musical characteristics, such as pulse clarity and spectral flux in low and high frequency components, influenced participants' movements, such as increased speed and more overall movement with high pulse clarity, whereas tempo of the music stimuli failed to exhibit any relationship to movement features. Moreover, Luck and Toiviainen (2006) as well as Luck and Sloboda (2008, 2009) investigated synchronization to conductors' gestures and found ensembles synchronize to maximal deceleration of the baton and that acceleration was best predicting the location of the beat along the movement trajectory.

Utilization of the body is further seen as the core concept of embodied cognition, which claims that the body is involved in or even required for cognitive processes (e.g., Lakoff and Johnson, 1980, 1999; Varela et al., 1991). Human cognition is highly influenced by the interaction between mind/brain, sensorimotor capabilities, body, and environment. Following this, we can approach music (or musical involvement) by linking our perception of it to our body movement and postulate that our bodily movements reflect, imitate, help to parse, or support understanding the structure of music (Leman, 2007). Leman suggests that corporeal articulations could be influenced by three (co-existing) components or concepts: *Synchronization*, *Embodied Attuning*, and *Empathy*, which differ in the degree of musical involvement and in the kind of action-perception couplings employed. *Synchronization* forms the fundamental component, as synchronizing to a beat is easy and spontaneous. The beat serves as the basic musical element, from which more complex structures emerge. Leman furthermore suggests the term *inductive resonance*, referring to the use of movements for active control, imitation, and prediction of beat-related features in the music (the opposite of passively tapping to a beat) as the first step in engaging with the music. The second component, *Embodied Attuning*, concerns the linkage of body movement to musical features more complex than the basic beat, such as melody, harmony, rhythm, tonality, or timbre. Finally, *Empathy* is seen as the component that links musical features to expressivity and emotions.

The present work investigates music-induced movement, in particular the aspect of moving in time with the music. It aims at studying how humans embody beat structure and periodicities related to metrical levels as well as entrainment to musical stimuli using full-body movement. Burger et al. (2013) established several relationships between musical characteristics and movement features; however, these features, while indicating relationships with pulse and rhythmic structure, were averaged across time and correlated with averaged movement characteristics, and therefore not applicable for analysis regarding underlying periodicities in the movement. Furthermore, no significant effect of tempo on the movement characteristics was found in the previous study, which raised the need for further investigation. Two experimental studies are presented in this paper. The first one deals with period locking to different metrical levels in full-body movement (unconstrained movement) and its relations to beat- and rhythm-related musical characteristics to address the shortage of research related to periodicity in music-induced movement. The other experiment deals more specifically with synchronization and phase locking in sideway swaying motions (constrained movement) to address the lack of research on phase locking in full-body movement and extend knowledge gained from tapping studies and the first experiment. This experiment can be seen as a follow-up to the first, being an attempt to decrease variability and degrees of freedom of the movement data, since the data that Experiment 1 yielded were found too unconstrained and complex for the precise investigation of accurate synchronization and phase-locking behavior.

A common hypothesis for both experiments—based on the dynamic attending theory (Jones, 1976; Drake et al., 2000)—was drawn from Toiviainen et al. (2010): vertical movement (constrained to bouncing up and down in Experiment 2) would be mostly embodied at the beat/tactus level, whereas horizontal movement (constrained to sideway swaying in Experiment 2) would be rather related to higher metrical levels, such as the bar (four beat) level. Furthermore, for Experiment 1, based on Burger et al. (2013), it was assumed that movement at tactus level, in particular in the superior-inferior (i.e., vertical) dimension, would be related to pulse clarity (i.e., period locking would increase with clearer pulse perceptions), presuming that pulse clarity measures the strength of the musical beat. Horizontal movement, in particular at bar level, on the other hand, could be more connected to rhythmic structures (as measured, e.g., by the spectral flux of certain frequency regions), as there is more freedom and flexibility to embody more complex structures than the basic beat in the horizontal plane as opposed to the vertical plane (e.g., due to gravity and biomechanical body properties). A third hypothesis related to Experiment 1 concerns the tempo: with increasing tempo, movement would occur period-locked to higher metrical levels, as biomechanical restrictions limit movement rates, or make them at least more effortful than moving at slower speeds. Furthermore, when taking the notion on preferred tempo into consideration (Fraisse, 1982; Moelants, 2002), it could be assumed that with tempos considerably slower than 120 bpm, people would use lower metrical levels, while with tempos faster than 120 bpm, people would rather use higher metrical levels to move at comfortable speeds.

Experiment 2 might be seen as a pilot study for analysis approaches to accurately investigating phase relationships between music and full-body movement. Based on findings that humans can detect regular beat sequences from metronomes and musical signals, and tap along with them in similar ways, it was firstly assumed that they would also sway their body in a common fashion to music. Thus, there would exist a common phase relationship between movement (e.g., a common point of change in movement direction) and beat location and that some kind of movement anchoring would happen (Beek, 1989). However, full-body movement is different, in particular more complex and unrestricted than finger tapping and therefore requires novel analysis methods, which will be elaborated in this paper.

## Experiment 1—period locking

### Materials and methods

#### Participants

Sixty participants took part in the study (43 female, 17 male, average age: 24, *SD* of age: 3.3). Six participants had received formal music education, while four participants had a formal dance background. Participation was rewarded with a movie ticket. All participants gave their informed consent prior to their inclusion in the study and they were free to discontinue the experiment at any point. Ethical permission for this study was not needed, which was according to the guidelines stated by the university ethical board.

#### Stimuli

Participants were presented with 30 randomly-ordered musical stimuli representing the following popular music genres: Techno, Pop, Rock, Latin, Funk, and Jazz. All stimuli were 30 s long, non-vocal, and in 4/4 time, but differed in their rhythmic complexity, pulse clarity, mode, and tempo. The stimulus length was chosen to keep the experiment sufficiently short while having stimuli that were long enough to induce movement. The list of stimuli is found in Burger (2013).

#### Apparatus

**Figure 1:**
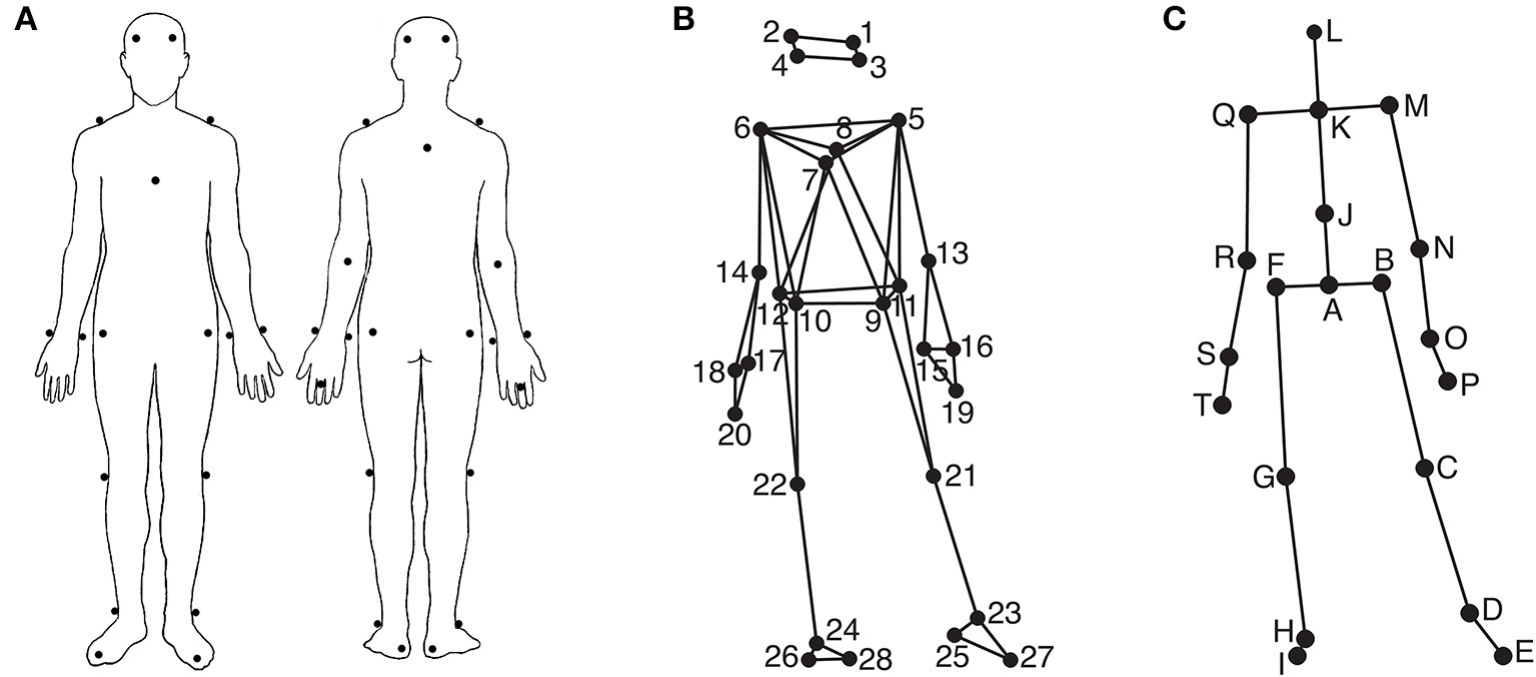
**Marker and joint locations. (A)** Anterior and posterior view of the marker placement on the participants' bodies; **(B)** Anterior view of the marker locations as stick figure illustration; **(C)** Anterior view of the locations of the secondary markers/joints used in the analysis.

Participants' movements were recorded using an eight-camera optical motion capture system (Qualisys ProReflex), tracking, at a frame rate of 120 Hz, the three-dimensional positions of 28 reflective markers attached to each participant. The locations of the markers can be seen in Figures 1A,B. The location of the markers were as follows (*L* = left, *R* = right, *F* = front, *B* = back): 1: LF head; 2: RF head; 3: LB head; 4: RB head; 5: L shoulder; 6: R shoulder; 7: sternum; 8: spine (T5); 9: LF hip; 10: RF hip; 11: LB hip; 12: RB hip; 13: L elbow; 14: R elbow; 15: L wrist/radius; 16: L wrist/ulna; 17: R wrist/radius; 18: R wrist/ulna; 19: L middle finger; 20: R middle finger; 21: L knee; 22: R knee; 23: L ankle; 24: R ankle; 25: L heel; 26: R heel; 27: L big toe; 28: R big toe. The musical stimuli were played back via a pair of Genelec 8030A loudspeakers using a Max/MSP patch running on an Apple computer. The room sound was recorded with two overhead microphones positioned at a height of approximately 2.5 m. This microphone input, the direct audio signal of the playback, and the synchronization pulse transmitted by the Qualisys cameras when recording, were recorded using ProTools software in order to synchronize the motion capture data with the musical stimulus afterwards. Additionally, four Sony video cameras were used to record the sessions for reference purposes.

#### Procedure

Participants were recorded individually and were asked to move to the presented stimuli in a way that felt natural. Additionally, they were encouraged to dance if they wanted to, but were requested to remain in the center of the capture space indicated by a 115 × 200 cm carpet^1^. The stimuli were presented after each other with a break of variable length in between, in which the participants were asked to rate the stimulus according to three questions (preference, familiarity, and movability). Participants were furthermore allowed to take breaks between the stimuli to drink or have a rest^2^.

### Movement data processing and periodicity analysis

Using MATLAB Motion Capture (MoCap) Toolbox (Burger and Toiviainen, 2013), the movement data of the 28 markers were first trimmed to 24 s between second 5 and second 29 to give the participants enough time to engage with the music in the beginning and to discard possible fade-out effects of the music in the end. Following this, the data was transformed into a set of 20 secondary markers—subsequently referred to as joints. The locations of these 20 joints are depicted in Figure 1C. The locations of joints C, D, E, G, H, I, M, N, P, Q, R, and T are identical to the locations of one of the original markers, while the locations of the remaining joints were obtained by averaging the locations of two or more markers; Joint A: midpoint of the four hip markers (called root in the further analysis); B: midpoint of markers 9 and 11 (left hip); F: midpoint of markers 10 and 12 (right hip); J: midpoint of breastbone, spine, and the hip markers (midtorso); K: midpoint of shoulder markers (manubrium), L: midpoint of the four head markers (head); O: midpoint of the two left wrist markers (left wrist); S: mid-point of the two right wrist markers (right wrist). Subsequently, the data was rotated, so that the hip joints (A, B, and F) were aligned to be parallel to the x-axis on average, and transformed to a local coordinate system with joint A being the origin. Next, the acceleration in three dimensions of the following nine joints B, D, F, H, K, M, O, Q, and S was calculated using numerical differentiation based on the Savitzky-Golay smoothing FIR filter (Savitzky and Golay, 1964) with a window length of seven samples and a polynomial order of two. These values were found to provide an optimal combination of precision and smoothness in the time derivatives.

**Figure 2:**
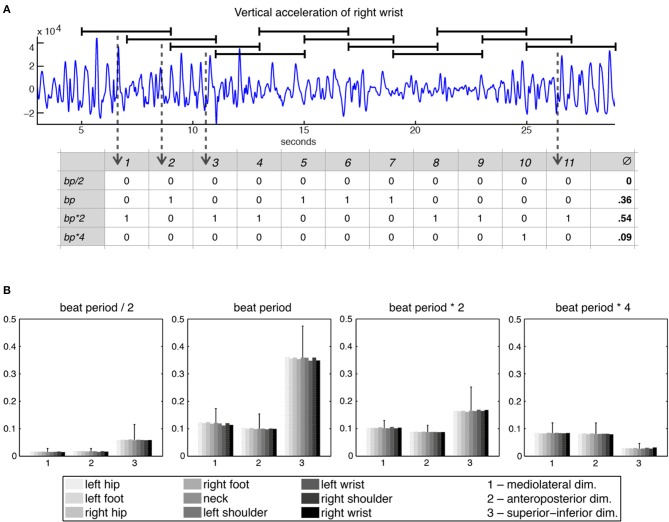
**Period-locking probability. (A)** Movement time series divided into 11 windows of 4 s each with a 2-s overlap and binary classification of the estimated periodicity per window with regards to four metrical levels; **(B)** Averaged period-locking probability per dimension and body part for each of the four metrical levels. The standard deviation (averaged across the body parts) is indicated by the vertical line on top of the bars.

Following these initial calculations, the 27 acceleration time series were divided into 11 windows of 4 s each with a 2-s overlap, with each window being separately subjected to periodicity estimation using auto-correlation (Eerola et al., 2006). Period locking per window was computationally evaluated by comparing the period estimate of the movement to four different metrical levels—half the beat period, beat period, two times the beat period, and four times the beat period—within a 5% tolerance. This approach resulted in a period-locking measure established by a quinary classification that assessed to which—if any—metrical level the participants were period-locked. Based on this classification (movement being either period-locked to one of the four metrical levels or not period-locked at all), a period-locking probability per metrical level was assessed by calculating the average of the period-locking occurrences per window and metrical level. Figure 2A shows the derivation of the period-locking measure, while Figure 2B shows the period-locking probability (averaged across participants) per metrical level, dimension, and body part.

For the three lower metrical levels (in particular for the beat level) the superior-inferior movement direction was found most often period locked, whereas for the bar level (four times the beat period) the horizontal dimensions were more often period locked. Since the four metrical levels showed a very consistent pattern across the different body parts, we decided to reduce the number of variables, taking the average across all body parts to produce one value per dimension for each metrical level.

### Musical feature extraction

To investigate the effect of beat- and rhythm-related features on periodic movement, we performed computational feature extraction analysis of the stimuli used in the experiment. Three musical features were extracted from the stimuli using the MATLAB MIRToolbox (version 1.4) (Lartillot and Toiviainen, 2007). The selection of features was based on features used in Burger et al. (2013).

Pulse Clarity: This feature indicates the strength of musical beats in the signal (see Lartillot et al., 2008).

**Figure 3:**
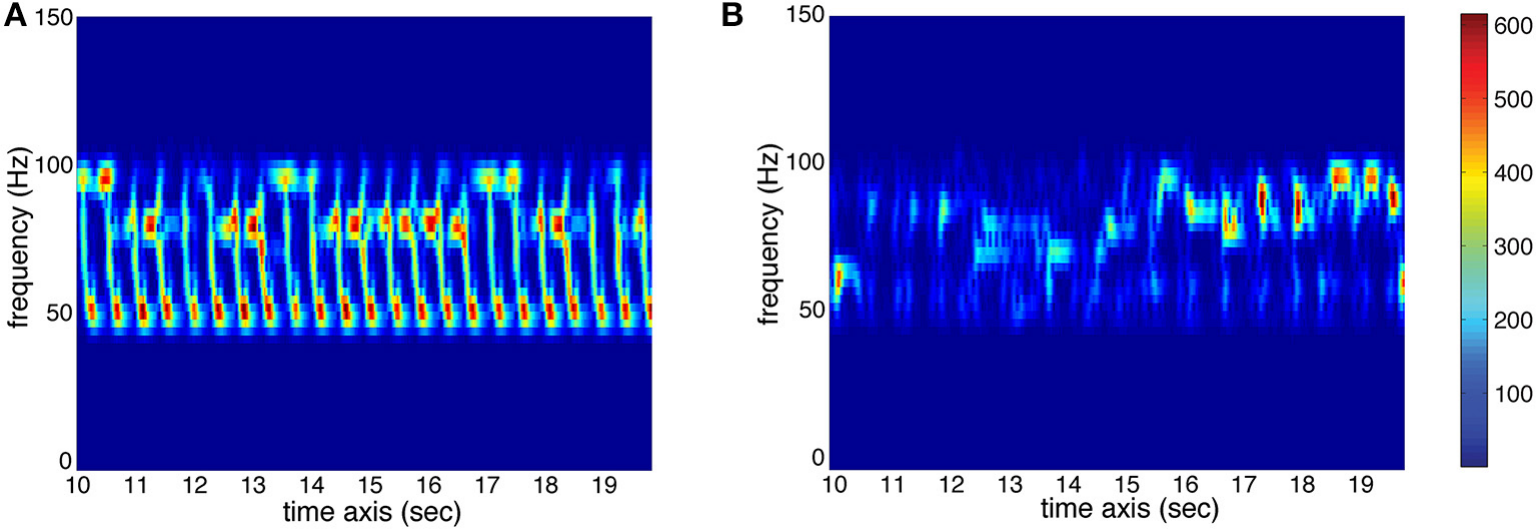
**Spectrograms (sec. 10–20) of sub-band no. 2 (50–100 Hz) of two stimuli used in the study. (A)** High amount of temporal change (red represents high energy at the respective time and frequency, whereas blue represents low energy; see color bar) resulting in high value for Sub-Band Flux. **(B)** Low amount of temporal change resulting in low Sub-Band Flux.

Low-frequency Spectral Flux: This feature indicates to which extend the spectrum changes over time. For the calculation, the stimulus is divided into 10 frequency bands, each band containing one octave in the range of 0–22,050 Hz. The sub-band flux is then calculated for each of these ten bands separately by calculating the Euclidean distances of the spectra for each two consecutive frames of the signal (Alluri and Toiviainen, 2010), using a frame length of 25 ms and an overlap of 50% between successive frames and then averaging the resulting time series of flux values. For the current analysis, we used sub-band no. 2 (50–100 Hz) to characterize Low-frequency Spectral Flux. High flux in the low frequency bands is produced by instruments such as the kick drum or bass guitar. Two spectrograms of sub-band no. 2 are displayed in Figure 3 to show the difference between high and low amounts of Sub-Band Flux.

High-frequency Spectral Flux: This feature uses the same calculation approach as the previous Low-frequency Spectral Flux feature. For the current analysis, we used sub-band no. 9 (6400–12800 Hz) to characterize High-frequency Spectral Flux. High flux in the high frequency bands is produced by instruments such as the cymbal or hi-hat.

In addition to these computationally extracted features, the Tempo of the stimuli was assessed in a separate tapping experiment, in which 10 participants tapped to the 30 stimuli. The tempo for each stimulus was determined by taking the median value of all intertap intervals. The tempo range was between 73 and 192 bpm (average 117.58, *SD* 25.62—the exact values for the stimuli can be found in Burger, 2013).

In order to ensure that the features measure different aspects of the stimuli, Spearman correlations were performed on the features. To account for the issue of multiple comparisons, false discovery rate control was utilized by applying the Benjamini-Hochberg procedure (see Benjamini and Hochberg, 1995) at a significance level of *p* = 0.05. As indicated in Table 1, only Low- and High-frequency Spectral Flux [ρ_(28)_ = 0.52, *P* < 0.01] moderately correlated with each other. However, since the same underlying algorithm is used to compute the spectral flux for the different sub-bands (that are furthermore derived from the same stimulus), correlations of this size are not regarded an issue.

**Table 1:** **Results of the (Spearman) correlation between the musical features**.

	**Low-frequency flux**	**High-frequency flux**	**Tempo**
Pulse clarity	0.37	0.02	0.05
Low-frequency flux		**0.52**	0.28
High-frequency flux			−0.19

*Significant correlations (p < 0.05) retained after applying Benjamini-Hochberg correction are marked in bold*.

### Results

In order to investigate how musical characteristics relate to periodic movement, we correlated the period-locking probability measure for each metrical level and movement dimension (averaged across body parts) with the four musical features. Due to the relatively large number of correlations, we utilized false discovery rate control by applying the Benjamini-Hochberg procedure (see Benjamini and Hochberg, 1995) separately for each metrical level at a significance level of *p* = 0.05. The results are presented in Table 2.

**Table 2:** **Results of the correlations between the period-locking probability measure and the musical features**.

	**Beat period/2**			**Beat period**		
	**ML**	**AP**	**SI**	**ML**	**AP**	**SI**
Pulse clarity	−0.05	−0.11	−0.04	**0.42**	**0.47**	**0.57**
Low frequency flux	−0.10	−0.13	−0.25	**0.63**	**0.58**	**0.54**
High frequency flux	0.10	0.04	0.21	**0.41**	0.39	0.30
Tempo	**−0.53**	−0.14	**−0.76**	0.06	0.03	−0.17
	Beat period * 2			Beat period * 4		
	**ML**	**AP**	**SI**	**ML**	**AP**	**SI**
Pulse clarity	0.45	0.46	0.03	0.18	0.19	−0.19
Low frequency flux	0.34	0.33	0.16	**0.55**	**0.55**	−0.08
High frequency flux	−0.05	−0.08	−0.32	−0.10	−0.08	−0.33
Tempo	0.32	0.26	**0.72**	**0.86**	**0.84**	**0.58**

*Significant correlations (p < 0.05) retained after applying Benjamini-Hochberg correction are marked in bold. ML, mediolateral; AP, anteroposterior; SI, superior-inferior movement dimension*.

Pulse Clarity showed significant positive correlations with all three movement dimensions at the tactus level (the beat period) [ML: *r*_(30)_ = 0.42, *p* < 0.05; AP: *r*_(30)_ = 0.47, *p* < 0.05; SI: *r*_(30)_ = 0.57, *p* < 0.01], suggesting that participants were period-locked in all dimensions to the tactus level when the music contained a clear beat structure.

Low Frequency Flux exhibited significant positive correlations with all three movement dimensions at the tactus level [ML: *r*_(30)_ = 0.63, *p* < 0.01; AP: *r*_(30)_ = 0.58, *p* < 0.01; SI: *r*_(30)_ = 0.54, *p* < 0.01] and with both horizontal dimensions at the bar level (four times the beat period) [ML: *r*_(30)_ = 0.55, *p* < 0.01; AP: *r*_(30)_ = 0.55, *p* < 0.01]. Thus, for stimuli with strong spectral flux in the low frequency range, the probability to be period-locked to these levels increased with increasing flux.

High Frequency Flux correlated significantly to the mediolateral movement dimension at the tactus level [ML: *r*_(30)_ = 0.41, *p* < 0.05], suggesting that participants moved mainly sideways within the beat period when the music had strong flux in the high frequencies.

Tempo showed a significant negative correlation with mediolateral and with superior-inferior movement at half the beat period [ML: *r*_(30)_ = −0.53, *p* < 0.05; SI: *r*_(30)_ = −0.76, *p* < 0.001], while correlations between superior-inferior movement and double the beat period [V: *r*_(30)_ = 0.72, *p* < 0.001] as well as both vertical and horizontal movement and four times the beat period [ML: *r*_(30)_ = 0.86, *p* < 0.001; AP: *r*_(30)_ = 0.84, *p* < 0.001; SI: *r*_(30)_ = 0.58, *p* < 0.01] were significantly positively related. These results suggest that period locking at half the beat period was more probable with slow stimuli, while period locking at higher metrical levels was more probable with faster stimuli.

## Experiment 2—phase locking

### Materials and methods

#### Participants

Thirty participants took part in the study (15 female, 15 male, average age: 28.2, *SD* of age: 4.4). Four participants had received formal music education and none had a formal dance background. Participation was rewarded with a movie ticket. All participants gave their informed consent prior to their inclusion in the study and they were free to discontinue the experiment at any point. Ethical permission for this study was not needed, which was according to the guidelines stated by the university ethical board.

#### Stimuli

Participants were presented with two Samba-style ballroom music stimuli. Both stimuli were 1 min long, with one having a tempo of 104 bpm (in the following, Stimulus 1) and the other one of 89 bpm (in the following, Stimulus 2). The stimuli were longer than in Experiment 1, as Experiment 2 was less about spontaneous engagement with the music, but participants were rather supposed to find and maintain a stable, non-changing movement pattern over the course of the stimulus presentation. To ensure non-ambiguous and stable tempos of the stimuli, a tapping experiment was conducted including eight participants. The distribution of medians, means, and standard deviations of the intertap intervals for both stimuli indicated that the participants were able to perceive the beat in the stimuli in a stable and consistent way.

#### Apparatus

Participants' movements were recorded using an eight-camera optical motion capture system (Qualisys Oqus 5+). The same tracking rate, marker setup, and playback and recording apparatus as in Experiment 1 were used.

#### Procedure

Participants were recorded individually, while being asked to sway sideways to the music. They were further encouraged to stay in one place and not to move too much besides the swaying motion.

### Movement data processing

As already described for Experiment 1, movement data were synchronized to the music and subsequently transformed to the joint representation using the MoCap Toolbox. Next, we rotated that data so that the hip joints (A and B) were aligned along (parallel to) the x-axis and extracted the root marker (joint A), on which the following analysis was performed. We restricted the analysis to the position of the root marker, since it reflects best any movement related to whole-body swaying and bouncing motions.

#### Periodicity analysis

**Figure 4:**
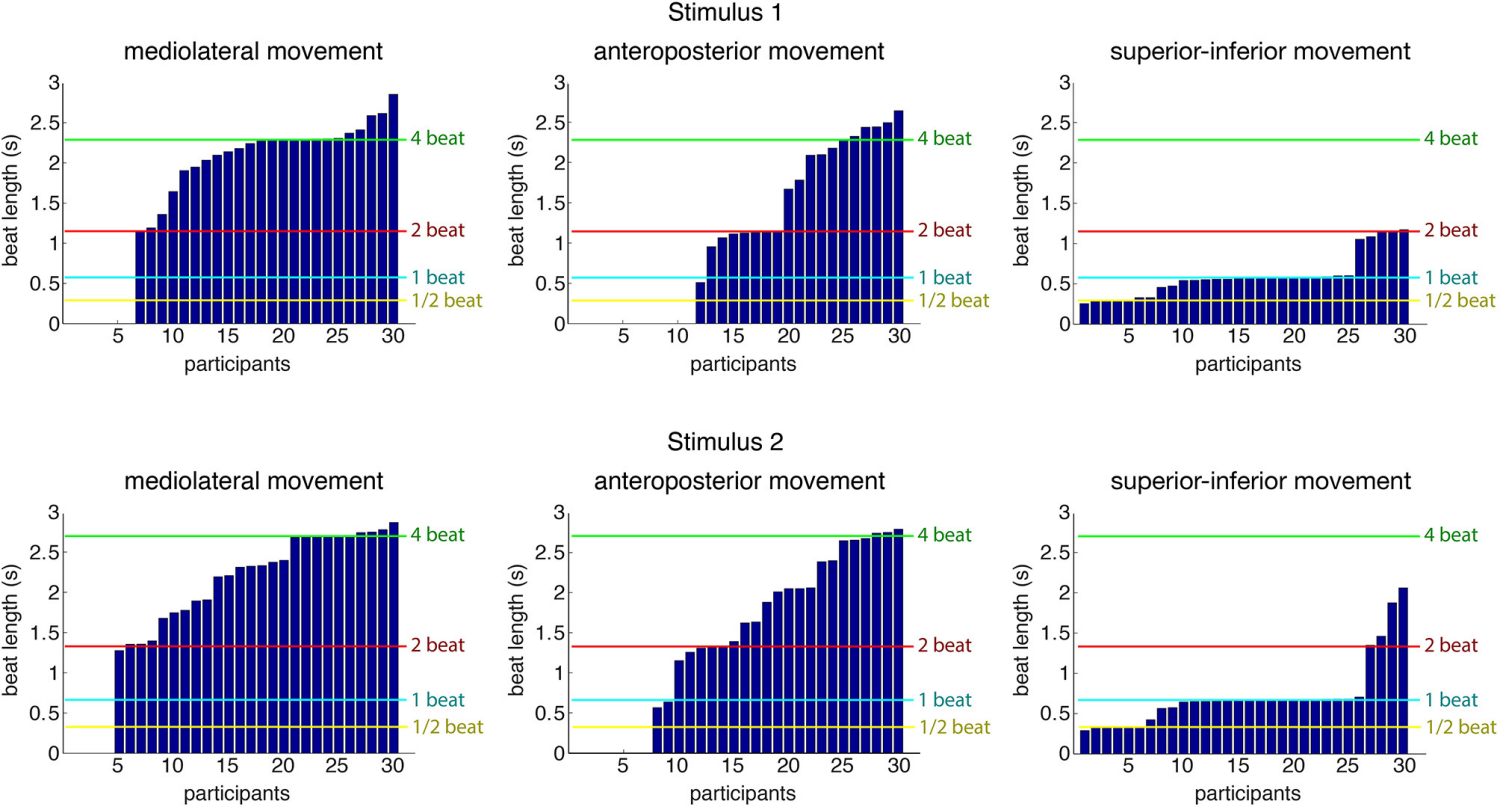
**Estimated movement periods of all participants (root marker) for the three movement directions**. Locations of four metrical levels (half the beat period, beat period, two times the beat period, and four times the beat period) are indicated for reference purposes in the plot. The data is sorted according to period estimates per stimulus and movement dimension, so participant numbers in the different graphs do not refer to same participant.

As a first step into the analysis, a periodicity analysis based on autocorrelation for the three movement directions was performed on the root marker, trimmed to a 30-s excerpt starting at second 20 of the original stimulus. This excerpt of the data was used to provide the participants with enough time to tune into the music and find a stable and periodic swaying pattern. Results regarding estimated movement periods of all participants for both stimuli can be found in Figure 4. Table 3 shows the number of participants being period-locked (within a 5%-tolerance of the beat period) to the four metrical levels for each movement direction and stimulus (a participant can only be locked to one—if any—metrical level).

**Table 3:** **Number of participants who were period locked within a 5%-tolerance for each of the four metrical levels**.

	**Stimulus 1**			**Stimulus 2**		
	**ML**	**AP**	**SI**	**ML**	**AP**	**SI**
Beat period/2	–	–	7	–	–	7
Beat period	–	1	**16**	–	1	**18**
Beat period * 2	2	5	4	4	5	1
Beat period * 4	**10**	2	–	**9**	6	–

*The two “metrical level—movement direction” combinations to which participants most often exhibited period-locked movement are indicated in bold*.

As can be seen, over half of the participants (stimulus 1: 16 of 30; stimulus 2: 18 of 30) were period-locked to the beat period in their superior-inferior movement, whereas about a third of the participants (stimulus 1: 10 of 30; stimulus 2: 9 of 30) were found to move periodically in mediolateral direction to the four beat level (bar level). Due to the low number of participants being period-locked to remaining metrical levels and movement dimensions, we decided to focus the subsequent analysis regarding phase locking on these two combinations of metrical levels and movement directions.

#### Phase-locking analysis

To address phase locking, the *phase of the movement* at metrical level *k* was estimated by band-pass filtering the root marker position with a zero-phase FFT filter at the frequency corresponding to the beat length of the metrical level, using a bandwidth of 15% of the center frequency, and subsequently applying a Hilbert transform. Next, we trimmed the data to 30-s excerpts starting at second 20. This yielded the movement phase relative to metrical level *k* as a function of time *t*, denoted by

$$\varphi_{k}(t)$$

Next, the time points of the musical beats in both stimuli were manually annotated using SonicVisualizer (www.sonicvisualiser.org). In order to compare the movement phase with the beat locations in the music, the *phase of the musical beat* at metrical level *k* was then estimated by linearly interpolating between the manually annotated beat locations. It is denoted by

$$\theta_{k}(t)$$

To assess the relationship between the movement and the musical beat, the *circular mean of the phase difference* at the respective metrical levels *k* was calculated by

$$re^{i\omega }=\frac{1}{t_{2}-t_{1}}\int_{t_{1}}^{t_{2}}e^{i\left(\varphi_{k}(t)-\theta_{k}(t)\right)}dt$$

with *k* indicating the metrical level and [*t*_1_, *t*_2_] denoting the time interval. This results in a vector whose length *r* indicates the strength/stability of the participants' phase locking and whose angle ω indicates the relation of the movement phase to the musical beat. To compensate for participants swaying in the same phase, but in opposite directions (i.e., by having started to sway either on the left or on the right side) for the mediolateral dimension/four beat metrical level, the circular mean was calculated relative to the movement phase being collapsed to a semicircle (i.e., multiplying the movement phase by two) and the two beat metrical level. Ultimately, the circular mean provides an estimate regarding the strength of participants' phase locking to the beat as well as its location in a circular space.

A way to visualize individual participants' phase-locking behavior can be achieved by using *Circular kernel density estimation* (Oliveira et al., 2012). *Kernel density estimates* (KDEs) of phase differences are calculated using the von Mises distribution, according to the formula

$$\hat{f}\left(\Phi \right)=\frac{1}{2\pi \left(t_{2}-t_{1}\right)I_{0}\left(\nu \right)}\int_{t_{1}}^{t_{2}}e^{\nu \left(\varphi_{k}\left(t\right)-\Phi \right)}dt,\;0\leq \Phi \leq 2\pi$$

where *I*_0_ (ν) is the modified Bessel function of order zero and ν is the concentration parameter.

### Results

**Figure 5:**
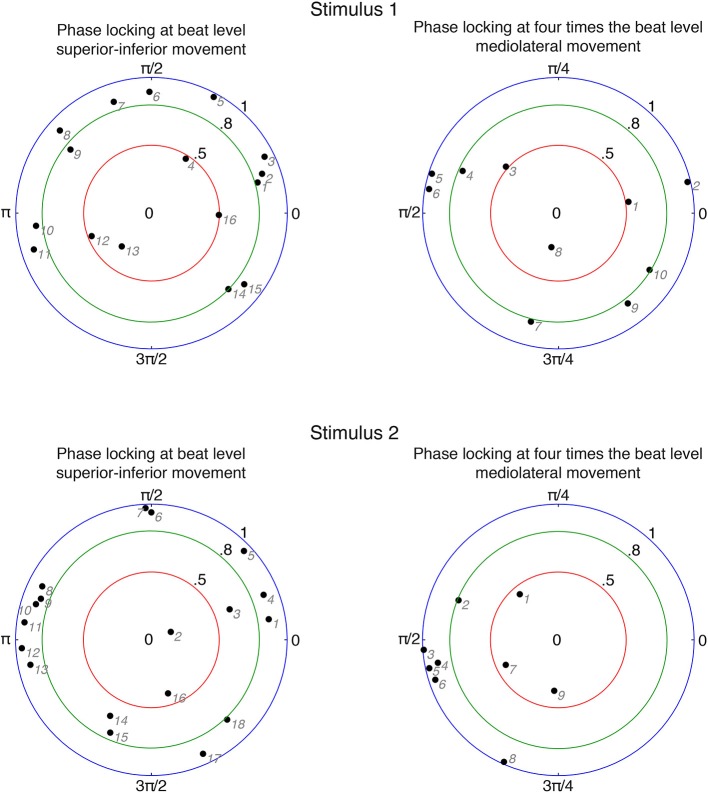
**Circular means of phase differences for period-locked participants**. Black numbers denote phase-locking strengths and angles. Gray numbers indicate individual participants ordered according to increasing angle (numbering corresponds to the phase-locked participants in Figure 6). Zero phase difference means that the movement is at an extreme position at the beat occurrence; for superior-inferior movement in particular it refers to the upper extreme position.

In order to investigate relationships between participants' movements and beat locations in the music, we calculated the circular mean of movement phases and beat locations for each participant. We only included participants who were period-locked within the 5% tolerance as described above. The results are displayed in Figure 5.

In case of superior-inferior movement at beat level, about 85% (stimulus 1: 13 out of 16; stimulus 2: 16 out of 18) of the period-locked participants showed a phase-locking stability of more than 0.5, and about 70% (stimulus 1: 11 out of 16; stimulus 2: 13 out of 18) exhibited a phase-locking stability of more than 0.8. These results indicate that most participants who moved in superior-inferior direction in a periodic fashion also moved with a very stable phase relation. However, participants failed to show a common relationship between the phase of their movement and the location of the beat, as the angles of the circular means were spread around the circle. There is, nevertheless, a slight cluster around 180° (π) visible for stimulus 2 (between #8 and #13), indicating that these participants changed from downwards to upwards movement at the occurrence of the beat.

For mediolateral movement at bar level (four times the beat), on average 78% (stimulus 1: 9 out of 10; stimulus 2: 6 out of 9) of the period-locked participants had a phase-locking stability of more that 0.5. About 53% (stimulus 1: 5 out of 10; stimulus 2: 5 out of 9) showed a phase-locking stability of more than 0.8, indicating that about half of the period-locked participants swayed sideways at bar level with a very stable phase. The angles of the circular means were less spread within the circle: for stimulus 1, they were located closer to the lower half of the circle, indicated that participants reached the extreme position in their swaying movement before the beat locations, however ranging from changing direction right on the beat to changing direction in the middle between two beats. For stimulus 2, the phase angles were even more narrowly distributed, mostly located at 90 (π/2), implying a somewhat common relation between movement phase and beat locations with changing the sway direction in the middle between two beats.

**Figure 6:**
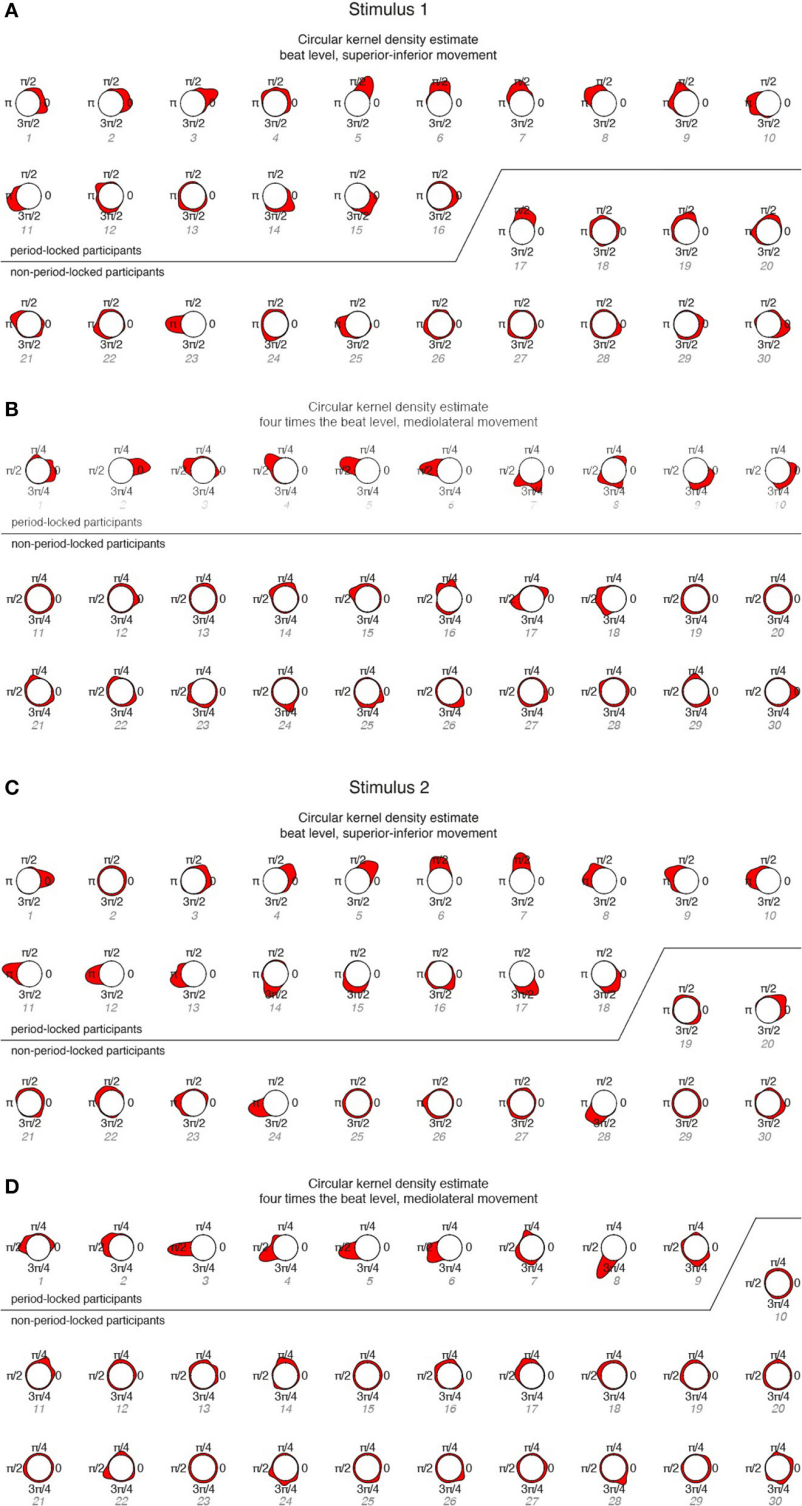
**Circular KDEs for each participant, ordered according to period-locked vs. non-period-locked participants and phase angle (numbering corresponds to Figure 5). (A)** Stimulus 1, beat level, superior-inferior movement; **(B)** Stimulus 1, four times the beat level, mediolateral movement; **(C)** Stimulus 2, beat level, superior-inferior movement; **(D)** Stimulus 2, four times the beat level, mediolateral movement.

To investigate participants' phase-locking behavior in more detail, the circular KDE for each participant was determined and displayed in Figure 6.

**Figure 7:**
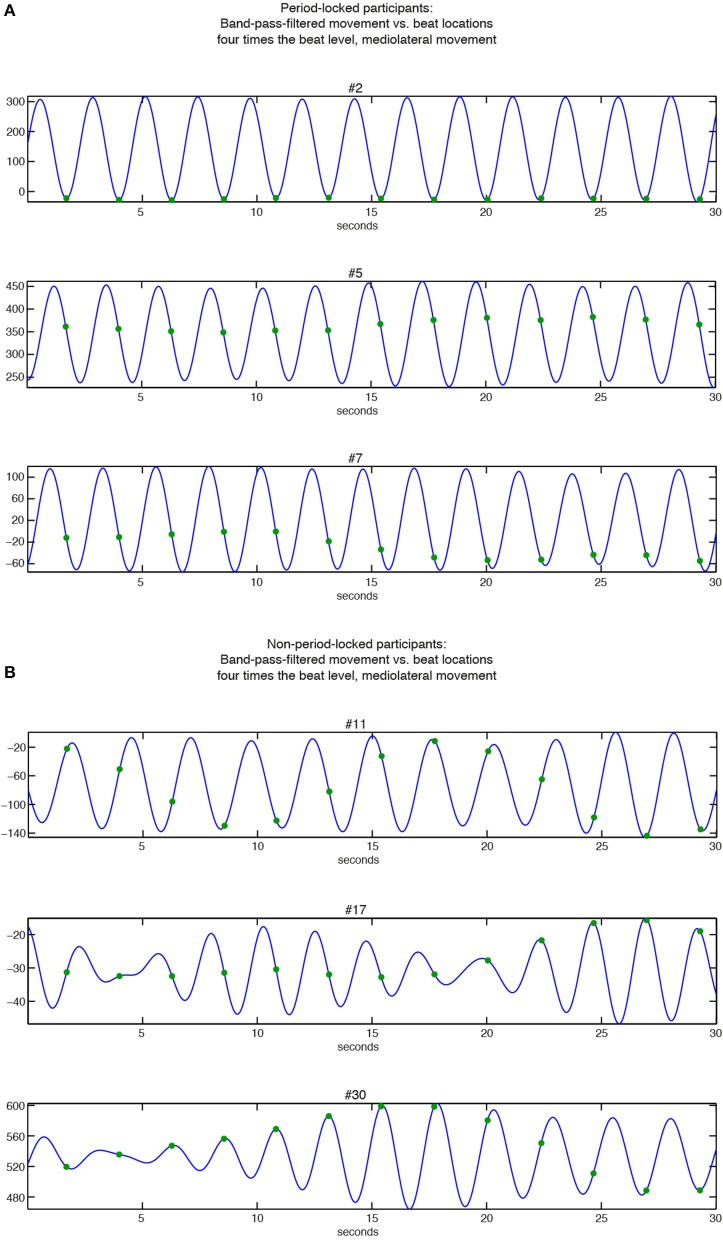
**Relationships between band-pass filtered position data of mediolateral movement (blue curve) and beat locations at bar level (green dots). (A)** Period-locked participants (#2, #5, and #7 in Figure 6B); **(B)** Non-period-locked participants (#11, #17, and #30 in Figure 6B).

The KDEs indicate both the strength of the phase locking (the length and the width of the kernel; i.e., longer and narrower = more stable the phase locking) and the location of the phase angle (the direction of the main peak of the KDE). For the period-locked participants, the KDE peak is clearly visible in most cases, whereas for the non-period-locked participants, there is, in most cases, no clear peak, but the KDE is instead spread around the circle, indicating a non-stable movement-beat relationship. Focusing on the mediolateral swaying movement more closely, Figure 7 displays several phase relationships of bandpass-filtered position data and location of musical beats for both period-locked (Figure 7A), and non-period-locked participants (Figure 7B).

The three participants displayed in Figure 7A show the diversity in swaying patterns ranging from changing movement direction right on the beat (#2), changing direction between two beats (#5), and changing direction before the beat occurrence (#7). As visible in Figure 6B, #7 showed two peaks in their KDE besides to each other, indicating a change in the phase relationship, which can be seen in the slightly moving movement-beat locations in the corresponding part of Figure 7A. This result also points to #7 being less strong phase-locked than #2 and #5 as indicated by the different vector lengths in Figure 5.

For the non-period-locked participants displayed in Figure 7B, the band-pass filtered data show more irregularities (especially for #17 and #30). Furthermore, the movement and the beat locations fail to establish a stable relationship, as there is a visible drift between them in all 3 examples.

However, for the non-period-locked participants, in some cases peaks are still clearly visible (e.g., #23 for stimulus 1—beat level, superior-inferior movement). This occurred in cases where the periodicity analysis resulted in a period estimate belonging to another metrical level than the one of interest for the particular analysis.

## Discussion

We conducted two experiments investigating period- and phase-locking behavior in music-induced movement. In both experiments, participants were asked to move to various (30–60 s) excerpts of real music. Experiment 1 allowed full-body dance-style movements which allowed us to assess main movement periodicities with regard to musical characteristics, whereas Experiment 2 constrained movements to sideway swaying, allowing us to focus our analysis on the precise phase relationships between movement and beat locations in the music.

The analysis for Experiment 1 showed that, first of all, participants exhibited period-locked movement at different metrical levels, and thus the whole body appeared to be entrained to a good extent when dancing to music. Furthermore, vertical movement was most often period-locked, in particular to the beat period (tactus level), whereas horizontal movement was found more often related to higher metrical levels. This finding supports our initial hypothesis and is furthermore in line with the results found by Toiviainen et al. (2010). It could be seen as being related to walking or swaying movements, requiring a longer evolvement of oscillatory motion. The relationship between movement direction (horizontal vs. vertical) and metrical level will be revisited when discussing results of Experiment 2, since this finding was among the primary considerations for conducting Experiment 2.

Concerning musical characteristics, Pulse Clarity was found to correlate significantly with the presence of periodic movement in all three dimensions at tactus level, supporting our hypothesis. At the tactus level, the basic pulse/beat structure should (naturally) be most salient. Moreover, music with strong and clear beats might be more inviting to move to, as the beat is easy to detect, affording quick and spontaneous entrainment (Burger et al., 2013). Movement in the superior-inferior direction exhibited the highest correlation value, which could suggest that clear pulses are most likely embodied by up and down movements, as it might be the most natural movement direction for movement period-locked to the beat level: superior-inferior movement can be executed fast enough to be entrained to the beat level, as gravity as well as muscular and biomechanical properties of the human body would support movement types such as bouncing up and down (as only the upward movement force is needed, since gravity effects the downward movement).

Along these lines, the significant correlations between periodic movement (in all dimensions), and Low Frequency Spectral Flux for the tactus and bar levels suggest relationships between rhythmic structures and at different metrical levels. Strong rhythmic structures in the low frequencies, such as those produced by kick drum and bass guitar, might encourage movement on different metrical levels simultaneously or interchangeably, since rhythmic structures are present on several metrical levels as well. Horizontal movements received higher correlations with Low Frequency Spectral Flux than vertical movement at the beat level, while additionally correlating with four times the beat period, which follows our hypothesis. A possible explanation for this finding could be that horizontal movements offer more possibilities for embodying periodicities related to rhythmic structures at different time scales, as these movement directions are more flexible and open to a variety of patterns than vertical movements, as they are less restricted by, for instance, gravity and muscular/biomechanical constraints. Regular up and down bouncing movements require more muscular tension and force when executed at slower speeds, thus they are less economical when performed periodically to bar level, whereas horizontal swaying movements would be more ergonomic and, at the same time, could represent more complex musical characteristics, such as rhythmic structures.

High Frequency Spectral Flux correlated significantly with period locking of mediolateral movement at the beat period. These results suggest that strong high frequency components, usually produced by hi-hat and cymbal sounds that create high-frequency rhythmical figures and hence affect the “liveliness” of the rhythm, appear to encourage humans to move sideways at the beat level to incorporate this liveliness. This result is somewhat in line and extends the results found in Burger et al. (2013) related to high frequency flux being embodied by head and hand movement (as well as the overall amount of movement).

As hypothesized, there is a progression visible in the correlation values between Tempo and the period-locking index: at low metrical levels the correlations were negative or around 0, whereas the higher the metrical levels, the more positive (higher) the values became. This suggests that participants tended to period-lock to lower metrical levels at slower tempi and to higher metrical levels at faster tempi. A possible explanation for this result could be that it was possible for the participants to move faster than the beat period when the tempo was slow, while for fast stimuli, it was impossible for participants to match the beat period, so they reduced the period of their movement to match the half bar (two-beat), and bar (four-beat) levels. Additionally, this result could be seen as support for the dynamic attending theory (Jones, 1976; Drake et al., 2000), according to which humans switch between metrical levels and attune to the one that fits best. It could further be related to the preferred beat period of 500 ms (Fraisse, 1982; Parncutt, 1994), in the sense that people might adapt their movement speeds to match the preferred tempo as closely as possible, thus using a lower metrical level when music is slower than 110/120 bmp and using a higher metrical level when music is much faster than that.

Experiment 2 went a step further in investigating entrainment behavior to music by examining phase locking between movement and beat locations in the music. First, a periodicity analysis was conducted, revealing that superior-inferior movement was most often period-locked to the tactus level (more than half the participants), while mediolateral movement tended to be period-locked to the bar level—however, mediolateral movement less frequently matching the period of the music than superior-inferior movement. This follows both our hypothesis and the results from Experiment 1 and is furthermore in line with Toiviainen et al. (2010). For anteroposterior movement, the periodicity pattern was less obvious, as participants were period-locked to different metrical levels. Both for this reason and as we were more interested in sideways swaying and up-and-down movements, we excluded anteroposterior movement from the further analysis and focused on superior-inferior movement at the tactus level and mediolateral movement at the bar level.

Analysis regarding phase locking of both combinations (superior-inferior movement/tactus level and mediolateral movement/bar level) produced a result that failed to support our hypothesis: the relationship between movement phase and beat locations was not consistent between participants, as the beat locations occurred at different phases of their movements. For both up-and-down and sideway swaying motion, people used rather heterogeneous and distinctive ways of embodying the beat in their movements. A possible explanation for these results might be found in individual differences, music or dance background, and (movement) preferences of different participants. Participants might nevertheless have anchored their movement to specific, but divergent, points in the music, which could imply that anchoring (Beek, 1989) to real musical stimuli is more complex than to metronomes or simple rhythmic stimuli (Keller and Repp, 2008), as real music might offer more possibilities for anchoring. Slight clusters of movement phase and beat location were obtained, in particular for the second stimulus, at π (superior-inferior movement), and π/2 (mediolateral movement), indicating that participants reaching the extreme position at the occurrence of the beat in case of superior-inferior movement, and on the back-beats in case of mediolateral movement. This specifically happened for stimulus 2, so one plausible explanation could be that the tempo of the stimuli (or other musical characteristics that differed between the two stimuli) had an effect on this behavior. However, this result is at odds with the notion of preferred tempo, as this would suggest that the stimulus closer to 110/120 bpm (i.e., stimulus 1) should have resulted in more coherent results.

A somewhat surprising result was that only about one third of the participants were able to entrain their sideway swaying motion in a stable manner with the music, despite being explicitly instructed to do so. One could imagine swaying to music as being a simple and low-level task, but results of this experiment suggest a more complex picture. It could be that the samba-style music used in the experiment might not have encouraged participants to strictly follow the instructions, but made them move somewhat more freely by adapting their motion to the musical characteristics (and in some cases, perhaps, incorporating samba-associated dance steps to some extent). Superior-inferior movement was found to be more frequently (slightly more than half of the participants) synchronized to the music (regardless of the metrical level) than mediolateral movement. It might be the case that bouncing up and down with the beat is a more natural movement direction when moving to music, or at least to this kind of music. Moreover, a possible explanation for this could be that bouncing movements synchronized to the beat period might be related to walking or stepping motions [(Fraisse, 1982); see Styns et al. (2007) for further connections between walking and beat patterns]. Furthermore, gravity and/or biomechanical properties/restrictions (as mentioned above) could play a role, as bouncing movements can be executed faster than swaying movements. Further studies will investigate whether humans find it more natural to bounce up and down or sway sideways to different styles of music, and which music and tempi enhance entrainment. Further analysis will also consider if background factors, such as music or dance training, have any effect on people's entrainment behavior.

The circular kernel density estimate offered an interesting way of visualizing data, making readily apparent synchronization differences amongst participants. The better phase-locked a participant was, the more focused the KDE tended to be. Nonetheless, the restriction to including only those participants that were period-locked to one metrical level made the problem apparent that participants who were period-locked to another metrical level might have still been phase-locked in a stable manner. A more refined analysis has to be developed to overcome this issue.

Linking our results to the broader framework of embodied music cognition (Leman, 2007), in particular to synchronization and inductive resonance, seems straightforward. The presence of a clear pulse structure not only encourages synchronization, but may also have a resonating effect on overall body movements that reflects the metrical structure and is what makes people entrain with the music. Thus, it could be claimed that metrical levels are embodied within the whole body, and not restricted to a certain body part. However, as outlined above, relations between movement directions and particular metrical levels seem to be linked to body/biomechanical properties and therefore embodied differently. Furthermore, taken the dispersion of (non)synchronization behaviors, periodic/synchronized movement to music might not be as simple and straightforward as one might expect—which could lead to the provocative notion that synchronizing to a musical beat may not be such a low-level process as usually claimed (Leman, 2007). Future research and analysis will be conducted on establishing more robust links between periodic movement and (beat locations in) music, including background factors, such as training and skill. One possible hypothesis could be that skilled dancers do not exhibit straightforward synchronization with the beat (and/or other metrical levels) due to their ability to move in more complex patterns than less skilled dancers.

Experiment 2 proved to be a constructive initial experiment for a possible attempt to phase-locking analysis, showing promising results. Nevertheless, different musical styles, and different tempi need further testing with this approach. Besides motion capture data, it would be very informative to collect additional data using force plates to gain insight into center of pressure and contact forces with the floor during swaying and bouncing motions. Such a study could reveal how the musical beat is embodied in terms of muscular activity. It might additionally be of interest to extend the experiment design to a more interactive, collaborative one that could investigate how people influence each other in their movement, and entrainment behavior. Furthermore, enlarging the experimental setup to incorporate brain measures would be valuable, as it would offer identification of neural correlates related to (bodily) entrainment.

We aimed at designing an ecologically valid study, in so far as this is possible with an optical motion capture system (which requires participants to wear a special clothing with markers), and in a laboratory situation. To this end, we chose real music stimuli (pre-existing songs of popular and common music styles), accepting the downside that they were less controlled, very diverse, and more difficult to analyze than artificially designed stimuli. However, this approach made it possible to present the participants with the kind of music that they were potentially familiar with and could have danced to. One could assume that this kind of music would make them move more and in a more natural fashion than more artificial stimuli.

The studies presented in this article investigated period- and phase-locking behavior in music-induced movement and reveal interesting findings regarding the ways humans entrain to music. These findings are still somewhat preliminary, as further research is required to more systematically pinpoint human entrainment with music including swaying, bouncing, and head nodding, the basic types of movement often exhibited when listening to music. Furthermore, it remains unclear what is actually meant by full-body movement being entrained to music, since full-body movement offers very unconstrained data with very many degrees of freedom. The suggested approaches of reducing analysis to averages across body parts or focusing on one marker seem to be useful paths, though it could still be beneficial to concentrate the analysis on individual body parts or derive a more comprehensive measure for “whole-body” movement. Moreover, the data representation used in such analyses is worth serious consideration, as location, velocity, or acceleration data might yield various insights, not to mention other possibilities of data processing, such as principal component analysis or calculations based on the kinetic energy of the movement. In conclusion, these studies imply that entrainment to music is a complex full-body behavior requiring further research.

### Conflict of interest statement

The authors declare that the research was conducted in the absence of any commercial or financial relationships that could be construed as a potential conflict of interest.

## Footnotes

^1^The exact wording of the instructions was: “Please move to the music how it feels natural to you. You may dance if you like.”

^2^Motion capture animations of movements performed to different stimuli can be found in the supplementary material of Burger et al. (2013).
